# Experimental Study of Dynamic Tensile Strength of Steel-Fiber-Reinforced Self-Compacting Concrete Using Modified Hopkinson Bar

**DOI:** 10.3390/ma16165707

**Published:** 2023-08-20

**Authors:** Jian Ma, Yifei Huo, Ning Wang, Zhang Sun, Liang Bian, Ruiyuan Huang

**Affiliations:** 1 School of Naval Architecture and Civil Engineering, Jiangsu University of Science and Technology, Zhangjiagang 215600, China; 2 Department of Modern Mechanics, University of Science and Technology of China, Hefei 230027, China; 3 Jiangsu Defeng Construction Group Co., Ltd., Zhangjiagang 215600, China; 4 College of Civil Engineering, Fuzhou University, Fuzhou 350116, China

**Keywords:** steel fiber, self-compacting concrete, dynamic tensile strength, modified Hopkinson bar

## Abstract

As a typical brittle material, the tensile strength of concrete is much lower than its compressive strength. The main failure mode of concrete buildings under explosive and impact loading is spalling, so it is crucial to understand the dynamic tensile performance of concrete. This paper presents an experimental study on the dynamic tensile strength of steel-fiber-reinforced self-compacting concrete (SFRSCC). Specimens of two different self-compacting concrete (SCC) mixes (C40 and C60) and four different fiber volume fractions (0.5%, 1.0%, 1.5%, and 2.0%) are fabricated. Dynamic tensile strengths of SFRSCC are obtained using a modified Hopkinson bar system. The relationships between the dynamic tensile strength of the corresponding SCC mix, the quasi-static compressive strength, and the fiber volume fraction are discussed. An empirical equation is proposed. It is shown that SFRSCC with high compressive strength has higher dynamic tensile strength than low-strength SFRSCC for the same fiber content, and the dynamic tensile strength of SFRSCC possesses an approximately linear relation with the fiber volume fraction. The mechanism underlying this fiber-reinforcement effect is investigated.

## 1. Introduction

Self-compacting concrete (SCC) finds applications in a large range of fields for its high flowability, moderate viscosity, and self-consolidation. The addition of steel fiber extends to the possible field of application of SCC as the concrete ductility and post-cracking resistance can be significantly improved; furthermore, the property of self-compactibility of SCC may also enhance the performance of the fibred composites by achieving a more uniform fiber dispersion compared with conventional fiber-reinforced concrete.

Until the present time, studies of steel-fiber-reinforced self-compacting concrete (SFRSCC) have mainly focused on its workability and quasi-static mechanical performance [1,2,3,4]. A variety of SFRSCC with different mix designs have been reported in the literature, and the influence of fiber types, fiber volume fraction, and aggregate size on the performance of SFRSCC have been investigated [5,6,7,8,9]. A regression model to determine the response surface of mixes proportioning of SFRSCC using statistical investigation was proposed by Kandasamy and Akila [10]. They designed a total of 30 mixtures. The fresh properties of SCC and mechanical properties of concrete were studied using Response Surface Methodology (RSM). Gulsan et al. [11] investigated the size effect on the compressive and tensile strengths of SFRSCC after exposure to high temperatures.

Concrete structures like protective shelters, nuclear reactor containments, offshore structures, and similar, may be subjected to high-strain-rate loading, caused by the impact of missiles or flying objects, also by vehicle collisions, or impulses due to explosions [12]; thus, it is necessary to understand the dynamic behavior of concrete. Habel and Gauvreau [13] used a drop-weight impact machine to study the dynamic fracture behavior of a self-consolidating ultra-high-performance fiber-reinforced concrete (UHPFRC). The results show a significantly increased strength and fracture energy of the dynamically loaded plates when compared to quasi-static loading. Caverzan et al. [14] used a modified Hopkinson bar (MHB) to perform the tensile tests on SFRSCC. It was observed that under the displacement rate of 1.2 m/s, the dynamic increase factor (DIF) on peak strength was 1.67. Cadoni et al. [15] investigated the dynamic behavior of self-compacting high-fiber-reinforced cementitious composite (HPFRCC) in tension when subjected to different strain rates. The strain rate sensitivity was studied using the dynamic increase factor. It is found that the DIF relationships proposed in the literature for plain concrete seem to be inadequate to describe HPFRCC. Zhang et al. [16] investigated the fracture behavior of three different SFRSCCs for a wide range of loading rates (strain rates from 10^−5^ to 10^1^ s^−1^) using both a servo-hydraulic machine and a drop-weight impact instrument. The results show that the flexural strength and the fracture energy are rate sensitive. At low loading rates, the rate effect is minor, while it is remarkable at high loading rates; moreover, with an increase in fiber content, the rate sensitivity is less. Lian et al. [17] conducted an experimental study of the dynamic compressive and tensile strengths of the basalt-fiber-reinforced alkali-activated concrete (BFRAAC). It is found that the strengths of BFRAAC material are significantly enhanced with the increase in strain rate. Compared to the compressive strength of the material, the strain rate sensitivity of its tensile strength is more marked. Wang et al. [18] investigated the dynamic properties of fly ash-slag-based strain-hardening geopolymer composites (SHGC) using a split-Hopkinson pressure bar. Results indicate that the strain rate had a significant influence on the compressive behavior of SHGC.

The experimental results on the dynamic behavior of SFRSCC so far are very scarce and the current understanding of the dynamic response, especially the dynamic tensile response of the material, is very limited. More tests are required for a better understanding of SFRSCC performance under dynamic loads and to assess the effects of different matrix strengths and contents of fibers on the dynamic tensile strength of SFRSCC under different loading rates.

In this paper, a new experimental method, based on the Hopkinson bar as a measuring device, was developed for the determination of the dynamic tensile strength of SFRSCC. Tests on SFRSCC specimens made of two different SCC mixes with different fiber volume fractions were performed. The relationships between the tensile strength, quasi-static compressive strength, and the fiber volume fraction of SFRSCC are obtained. The fiber-reinforcement effect on the tensile strength of SFRSCC was also discussed.

## 2. Experimental Procedure

### 2.1. SFRSCC Mix Design

**Figure 1:**
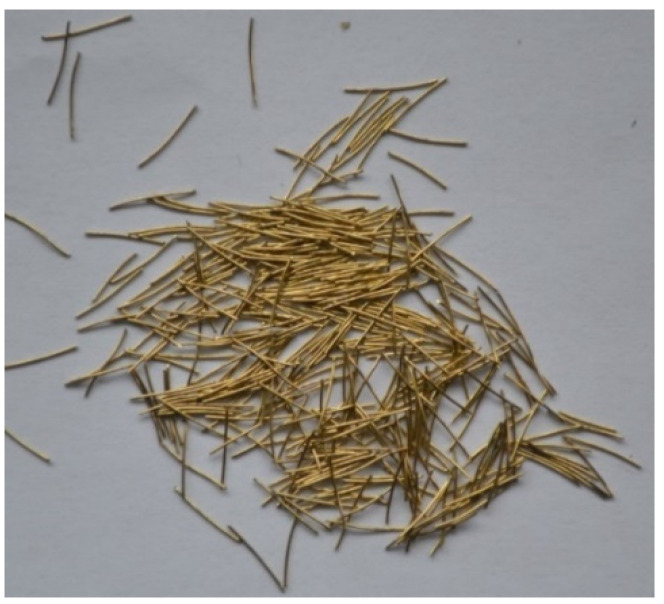
Steel fibers used in this study.

The investigations were carried out on two different SCC mixes: C40 and C60. For each SCC mix, four different volume fractions of steel fiber were added to cover the majority of practically used fraction ranges: 0.5%, 1.0%, 1.5%, and 2.0%. The constituent materials used in the composition of SFRSCC were: ordinary Portland cement (42.5R), fine river sand (2.39 fineness modulus), continuously graded limestone coarse aggregate, fly ash, water, superplasticizer based on polycarboxylates, and straight high-carbon-steel fibers. The SFRSCC mix proportions were determined based on the standards of JGJ/T 283-2012 [19] and JGJ/T 221-2010 [20]. Table 1 shows detailed mixture composition. Table 2 shows the continuous gradation of coarse aggregates. The steel fibers are shown in Figure 1, whose yield strength is 780 MPa according to the supplier, and are characterized by a diameter equal to 0.2 mm, a length equal to 10 mm, and an aspect ratio equal to 50.

**Figure 2:**
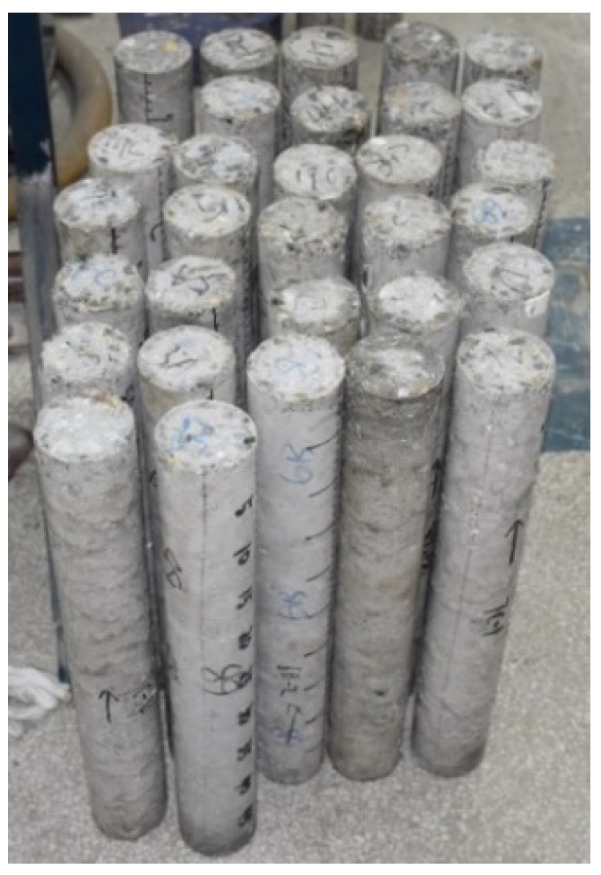
Part of the SFRSCC specimens.

For each type of SFRSCC, five specimens of 71 mm diameter and 500 mm length were fabricated for spalling tests. Figure 2 shows part of the specimens. Because of the self-compacting property of SFRSCC, the fabrication of specimens in this study was more convenient than that for traditional concrete. No vibration and coring was needed. SFRSCC cylinders were directly cast in molds of PVC tubes of 75 mm inner diameter and 520 mm length. These cylinders contained in PVC tubes were wet-cured for 30 days at room temperature. Then, PVC tubes were peeled away from SFRSCC cylinders, and two base surfaces and the side surface of specimens were ground to ensure that the specimens possess a size of 71 mm diameter and 500 mm length. The two ends were ground plane, smooth, and parallel, in order to ensure contact of pressure-bar/specimen interfaces and reduce friction effects. As shown in Figure 2, there are rarely air holes on specimens’ surfaces, demonstrating the good compactness of SFRSCC.

### 2.2. Basic Properties of SFRSCC

To ensure its self-compacting property, SFRSCC mixes must possess certain workability properties, which mainly include filling ability, passing ability, and segregation resistance. The filling ability ensures that SFRSCC can fully populate molds; the passing ability ensures that SFRSCC can pass through rebar intervals; and the segregation resistance prevents separation of aggregates and fibers from cement mortar. Several test methods are commonly used to determine workability properties of SCC, namely, slump-flow test, L-box test, slump-flow time test, U-box test, and J-ring test. These test methods and corresponding properties are shown in Table 3. Slump-flow tests by Abram’s cone were performed to determine the filling ability and segregation resistance of SFRSCC. As shown in Table 4, the slump-flow values of eight types of SFRSCC mix are in the range of 710–845 mm, demonstrating acceptable filling ability for practical applications. Throughout slump-flow tests for all types of SFRSCC mix, no signs of segregation were detected, and the mixture maintained good homogeneity and cohesion. L-box tests were adopted to examine the passing ability of SFRSCC. Table 4 shows the $h_{2}/h_{1}$ values of SFRSCC mixes. The $h_{2}/h_{1}$ values are in a practically acceptable range: 0.89–0.94.

The compressive strengths of SFRSCC were evaluated using quasi-static compressive tests on 150 mm cubes at 28 days. Table 4 includes the compressive strengths of a total of eight types of SFRSCC. As shown in Table 4, SFRSCCs based on C40 SCC possess strengths from 43.9 to 49.8 MPa, and C60 from 60.1 to 69.6 MPa, both matching the strength requirements of corresponding grades. One thing to note, quasi-static compressive strength of SFRSCC for the same SCC mix does not increase with the increase in fiber content. The reason is that the mixture proportions were modified with variations in fiber content (as shown in Table 1) to ensure the workability and self-compaction of SFRSCC.

### 2.3. Modified Hopkinson Bar Technique

**Figure 3:**
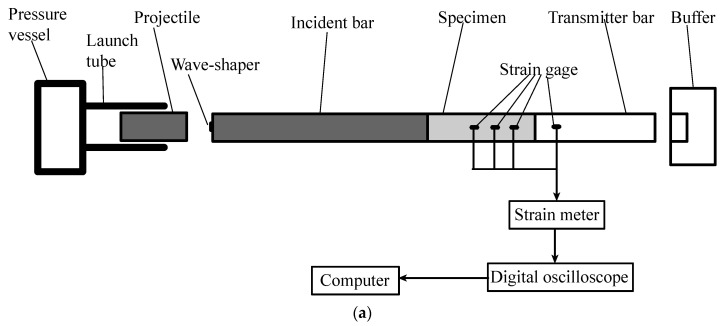

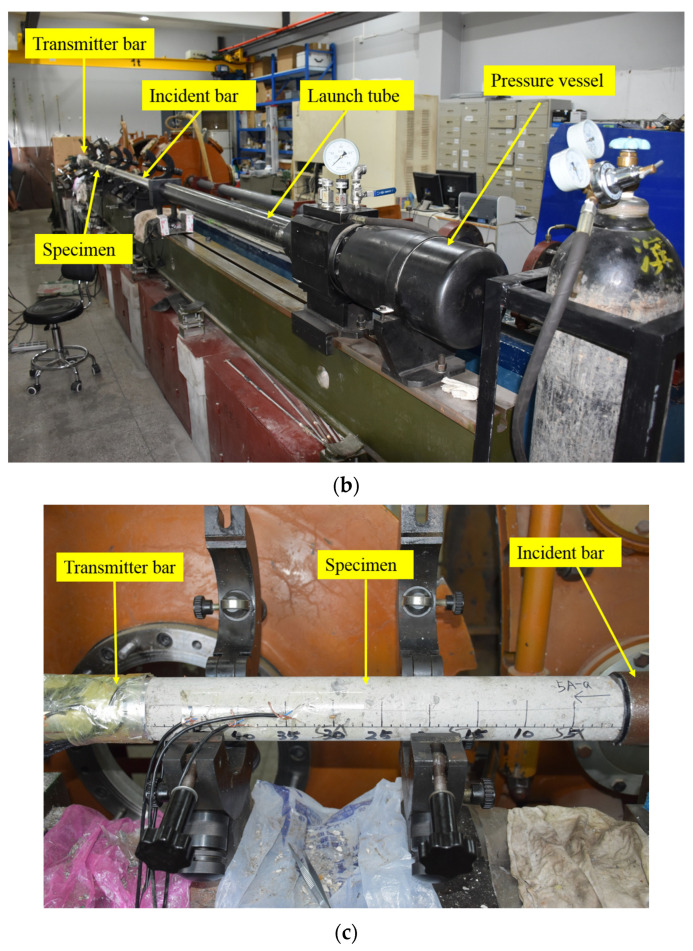
Setup of modified Hopkinson bar: (**a**) schematic diagram of the equipment; (**b**) photo of the equipment; (**c**) specimen arrangement.

The authors adopted modified Hopkinson bar technique, proposed and improved by Hu et al. [21] and Zhang and Hu [22], to determine dynamic tensile strength of SFRSCC. Figure 3 shows this facility schematically. The test system consists of a steel cylindrical projectile, a steel cylindrical incident bar, an SFRSCC cylindrical specimen, an aluminum cylindrical transmitter tube, and a buffer. To ensure arising of tensile stress, $\frac{l_{s}}{C_{s}}$ should be greater than $\frac{l_{p}}{C_{p}}$ where $l_{p}$ and $l_{s}$ are lengths of projectile and specimen; $C_{p}$ and $C_{s}$ are elastic wave velocities in projectile and specimen. In this study, the projectile of 75 mm diameter and 300 mm length was launched from a gas gun, which impacted the strike-end of the incident bar, where a compressible wave-shaper was placed. The incident bar was 75 mm in diameter and 3500 mm in length, and the transmitter tube had a 60 mm inner diameter, 70 mm external diameter, and 1000 mm length. The specimen, with Vaseline applied evenly over two ends to reduce friction effects, was sandwiched between the incident bar and transmitter tube. The transmitter tube was employed to ensure that its equivalent wave impedance was much lower than the specimen’s. Three pairs of strain gages were attached to SFRSCC specimen’s surface with a 50 mm distance between each other, to measure stress pulse signals in the specimen. These signals were used to calculate each SFRSCC’s wave speed. One pair of strain gages were attached to transmitter tube’s external surface at the location of 50 mm to specimen\transmitter-tube interface, to measure stress pulse signal in the transmitter tube. These signals were used to detect spalling signs and determine each specimen’s tensile strength.

**Figure 4:**
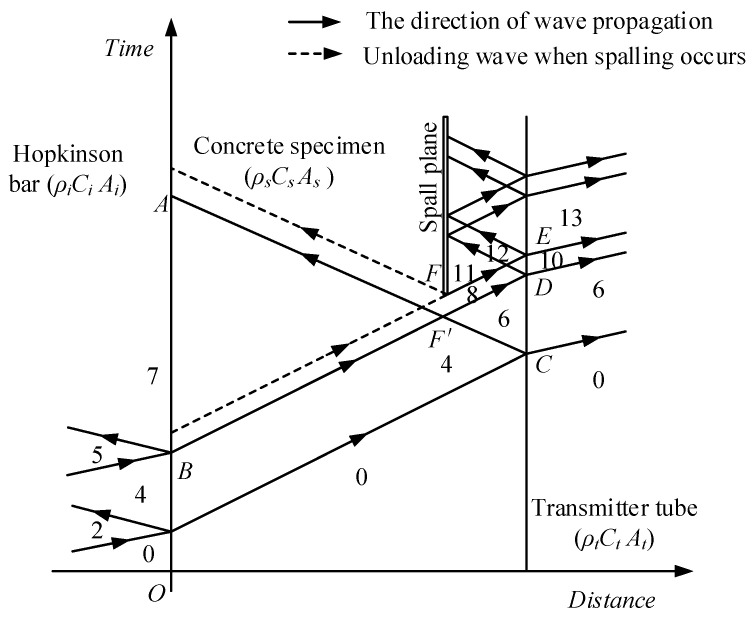
Time–distance diagram of wave propagation.

**Figure 5:**
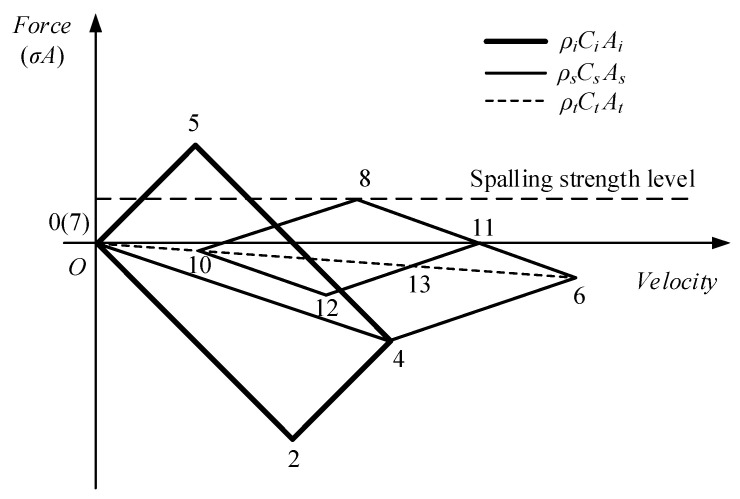
Force–velocity diagram of wave propagation.

**Figure 6:**
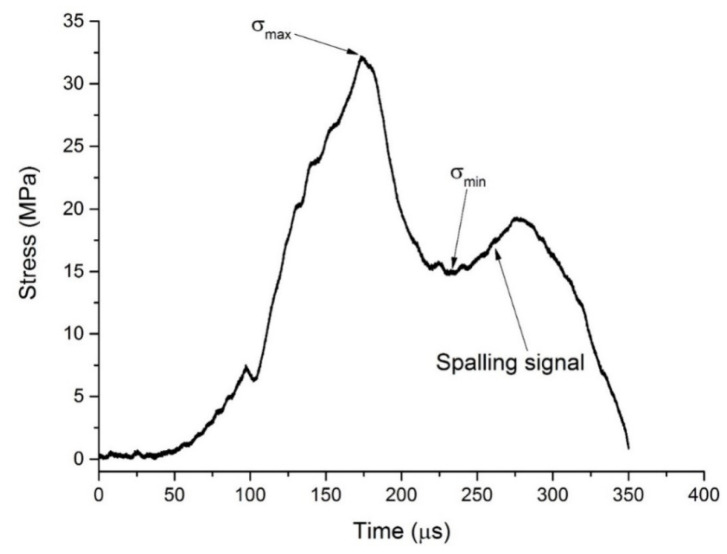
Stress pulse profile in the transmitter tube.

Figure 4 and Figure 5 show the propagation of waves in bar system and the arising of spalling in specimen. The projectile impacts on incident bar and generates a compressive stress wave, which propagates along incident bar to the incident-bar\specimen interface. The pulse is partly transmitted through the interface (State 4 in Figure 5) and travels along the specimen to the specimen\transmitter-tube interface. At this interface, the compressive pulse is reflected to generate a tensile pulse propagating to the specimen because of the comparatively low equivalent-wave impedance of the transmitter tube. This tensile pulse encounters the other one reflected from free surface of projectile at $F^{\prime }$ point, as shown in Figure 4. Tensile stress state arises at the time and position represented by $F^{\prime }$. At F point, tensile stress exceeds the spalling strength of the specimen, and tensile damage accumulates until the specimen cracks into two pieces and generates a free surface. At the free surface, the tensile pulse is reflected to generate a compressive pulse, which unloads the tensile pulse to zero (State 11 in Figure 5) and is regarded as spalling signal. Sometimes, the tensile pulse is so large that spalling happens several times and the specimen cracks into a couple of pieces. All the propagation and reflection of pulse that happens in specimen can be reflected by the stress history at the specimen\transmitter-tube interface, which is represented by the stress pulse measured using the strain gages on transmitter-tube with acceptable accuracy. Assuming that both SFRSCC and the tube aluminum are linear elastic and that all the pulses are one-dimensional, the tensile strength $\sigma_{t}$ (i.e., spalling strength) can be calculated using the following formula [22]:

(1) $$\sigma_{t}=\frac{1}{2}\left[\left(\sigma_{max}+\sigma_{min}\right)-\frac{K_{s}}{K_{t}}\left(\sigma_{max}-\sigma_{min}\right)\right]\frac{A_{t}}{A_{s}}$$

where $\sigma_{max}$ is the peak value of the interface stress history, and $\sigma_{min}$ is the minimal value of the interface stress history before the spalling signal, which are illustrated in Figure 6; $K_{s}=\rho_{s}C_{s}A_{s}$ and $K_{t}=\rho_{t}C_{t}A_{t}$ are the equivalent wave impedances of specimen and transmitter tube; $\rho_{s}$ and $\rho_{t}$ are densities of SFRSCC and aluminum; $C_{t}$ and $C_{s}$ are wave velocities of SFRSCC and aluminum; $A_{t}$ and $A_{s}$ are cross-sectional areas of SFRSCC and aluminum.

There are several modifications in the technique used in this study compared with Hu and Zhang’s. Firstly, they use a linear tapered projectile to produce a half-sine incident pulse [21,22,23], which is comparatively costly as a specifically designed projectile must be manufactured for a specific material. We used a cylindrical projectile with a brass wave-shaper placed at the center of the strike-end of incident bar to generate half-sine incident pulse [24,25]. This method is relatively cheap and was verified by Sun et al. [26]. Secondly, Hu and Zhang used nylon rod as transmitter bar, whose visco-elastic property reduces experimental precision and makes signal processing more complex [22]. We adopted aluminum tube that is linear-elastic and guarantees a low equivalent wave impedance as well.

## 3. Results and Discussion

### 3.1. Experimental Results

**Figure 7:**
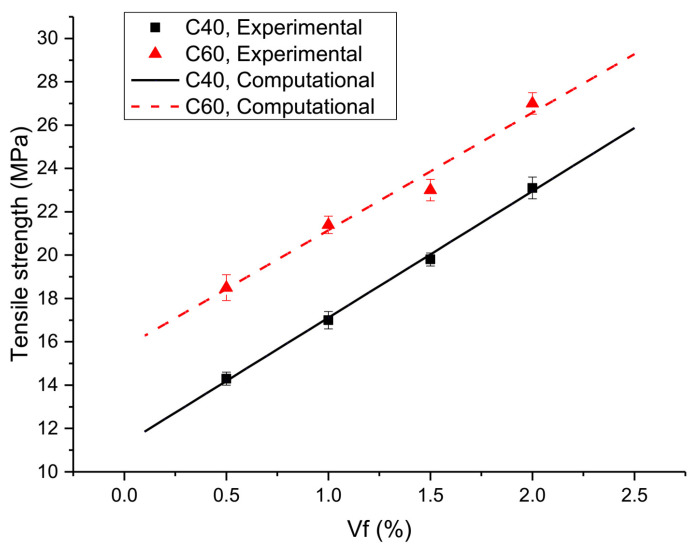
Dynamic tensile strength of SFRSCC.

For each type of SFRSCC, five specimens were fabricated for the spalling tests. At least three successful tests were conducted and the average result was used for analysis. For each test, the projectile’s impact velocity was set to 8 m/s, and the stress rate was approximately 0.65 TPa/s, which is estimated by dividing the peak stress of the stress pulse in the transmitter tube by the duration of the same stress pulse’s loading section. The tensile strengths of a total of eight types of SFRSCC are listed in Table 5 and shown in Figure 7. An empirical equation is developed to express the relationship between the SFRSCC’s tensile strength and its quasi-static compressive strength and fiber volume fraction:

(2) $$\sigma_{t}=2.35+6.68V_{f}+0.223f_{c}-0.021V_{f}f_{c}$$

Figure 7 compares the experimental data and empirical curve, showing a good consistency between them. It is notable that due to the limited experimental data, Equation (2) reflects the tensile strength pattern in a finite scope.

Four conclusions are drawn from the results. First, the dynamic tensile strength of SFRSCC is affected by the quasi-static compressive strength of the corresponding SCC mix. With the same fiber content, the C60-class SFRSCCs possess higher dynamic tensile strengths than the C40-class SFRSCCs. The conclusion is similar to the quasi-static tensile test results on plain concrete [27], which suggest that the compressive strength of SCC plays a similar role in both SFRSCC dynamic tensile strength and SCC quasi-static tensile strength.

Secondly, the dynamic tensile strength of SFRSCC possesses an approximately linear relation with the fiber volume fraction, similar to the phenomena observed on steel-fiber-reinforced concrete by Zhang et al. [28]. As shown in Figure 7, for each SCC mix, the four data points are approximately in a straight line.

Thirdly, with the increasing steel fraction, tensile strength increases slightly slower for SFRSCC with higher quasi-static compressive strength, which is represented by the fourth item on the right side of Equation (2), and vice versa.

Other than the last two factors, for each SCC mix, the compressive strengths of SFRSCC show no observable effect on the dynamic tensile strength of SFRSCC. As shown in Figure 7, for each SCC mix, dynamic tensile strengths of SFRSCC possessed an approximately linear relation with the fiber volume fraction, with sightless bias caused by a variation in the quasi-static compressive strength of SFRSCC (listed in Table 4).

### 3.2. Fiber-Reinforcement Effect on SFRSCC Tensile Strength

The fiber-reinforcement effect on the dynamic tensile strength of SFRSCC is evident. The underlying mechanism is explained based on energy consumption. In the process of tension and crack of concrete, micro-cracks in it generate, develop, and eventually concentrate to make macro-cracks, which make for the fracture of concrete [29]. The fiber pull-out effect plays an additional role in the tension of SFRSCC. Steel fibers are randomly distributed within SFRSCC and strongly bonded with the matrix. So, when the developing micro-cracks encounter fibers in SFRSCC, the fibers will be pulled out from the matrix. First of all, pulling fibers out from the matrix itself consumes extra energy, making SFRSCC tougher to resist tensile forces than plain concrete. Meanwhile, the pull-out of fibers defers the composition of macro-cracks and, therefore, contributes to the generation of extra micro-cracks and the complication of micro-crack developing paths, which consume additional energy as well. In conclusion, the addition of steel fibers defers the development of micro-cracks and the transition from micro- to macro-cracks by consuming additional energy; therefore, the tensile strength of SFRSCC increases with the increase in fiber content.

**Figure 8:**
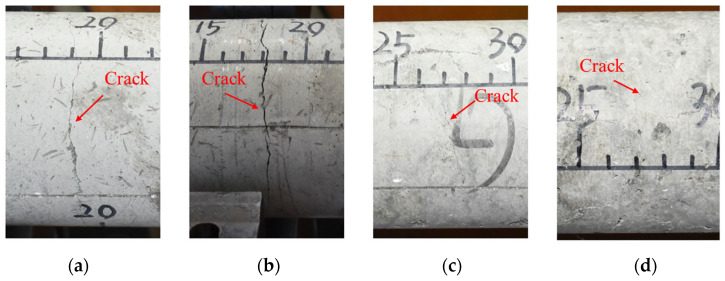
Fracture patterns of SFRSCC with different fiber volume fractions: (**a**) C60-0.5%; (**b**) C60-1.0%; (**c**) C60-1.5%; (**d**) C60-2.0%.

**Figure 9:**
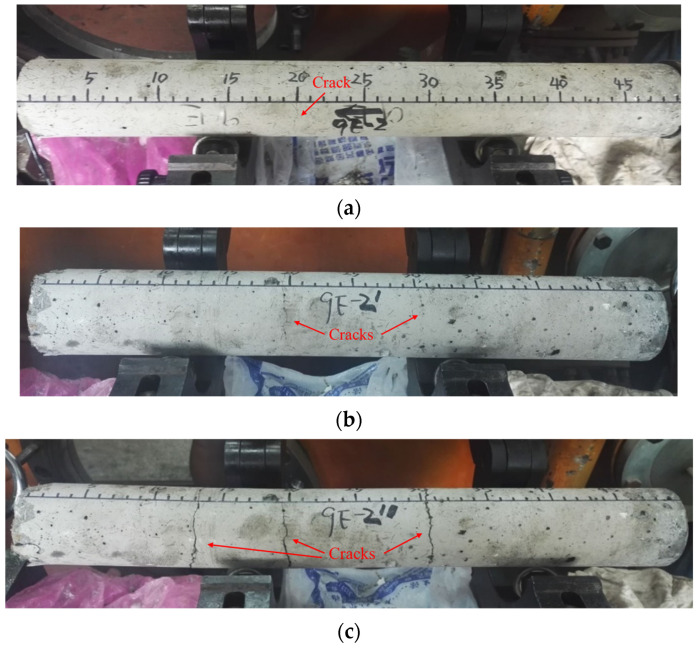
Multiple loading on the same specimen (C60-0.5%): (**a**) First loading; (**b**) Second loading; (**c**) Third loading.

Fracture patterns of the SFRSCC with different fiber volume fractions are shown in Figure 8. As can be seen, the cracks, which are distributed in the entire circumference of the concrete surface, are obvious when the fiber content is relatively low. Due to the bonding effect of steel fibers, the specimen is not completely separated along the fracture plane. With the increase in fiber content, the cracks become finer, and the distribution of the cracks is discontinuous, which indicates that most of the tensile load in the specimen is carried by the steel fibers during the spalling test. The variations in quantity and width of the cracks under multiple loading on the same specimen are shown in Figure 9. It can be observed that SFRSCC can withstand multiple loading and the number and width of cracks increase with the increase in loading times. The specimen is divided into four sections, which are still not separated from each other after three times of loading. The results indicate that SFRSCC has better resistance to repeated loading than plain concrete. This can be helpful for the design of military protection structures, which may suffer multiple attacks.

## 4. Conclusions

The dynamic tensile strength of steel-fiber-reinforced self-compacting concrete is studied using modified a Hopkinson bar. SFRSCC specimens, with different SCC mixes and different fiber contents, were manufactured. Basic tests were performed on them to determine their workability and compressive strength. The Hopkinson bar technique proposed by Zhang and Hu (2006) was adopted and modified to determine the SFRSCC’s dynamic tensile strength more conveniently and accurately.

The experimental results indicate that the tensile strength is mainly related to the compressive strength of the corresponding SCC and the fiber volume fraction. The dynamic tensile strength of SFRSCC increases with the increase in static compressive strength and fiber volume fraction. An empirical equation is developed to express the relationship between the SFRSCC’s tensile strength and its quasi-static compressive strength and fiber volume fraction. The mechanism underlying the fiber-reinforcement effect is discussed. We think the pull-out of steel fibers reduces crack development within the concrete and, therefore, improves the tensile strength of SFRSCC.

## Figures and Tables

**Table 1:** Mix proportions of SFRSCC (kg/m^3^).

Specimen Type	Cement	Sand	Aggregate	Fly Ash	Water	Superplasticizer	Fibers
C40-0.5%	427	724	862	107	171	2.6	39
C40-1.0%	427	721	856	107	171	2.6	78
C40-1.5%	427	714	849	107	171	2.6	117
C40-2.0%	427	708	843	107	171	2.0	156
C60-0.5%	494	714	831	49	148	3.3	39
C60-1.0%	494	696	810	49	148	4.4	78
C60-1.5%	494	678	789	49	148	5.1	117
C60-2.0%	494	658	778	49	148	3.6	156

**Table 2:** Percentage of passing sieves having square openings.

Mesh Size (mm)	Passing Percentage (%)
2.36	98.43
4.75	93.42
9.5	44.68
16	2.13
19	0.00

**Table 3:** Test methods and corresponding properties of SFRSCC.

Test Method	Properties Tested
Slump-flow test	Filling ability, Segregation resistance
L-box test	Passing ability, Segregation resistance
Slump-flow time test	Filling ability
U-box test	Passing ability, Segregation resistance
J-ring test	Passing ability

**Table 4:** Basic properties of SFRSCC.

SFRSCC Type	Slump-Flow Value (mm)	L-Box Test (*h*_2_/*h*_1_)	Quasi-Static Compressive Strength *f_c_* (MPa)
C40-0.5%	795	0.91	49.8
C40-1.0%	845	0.91	43.8
C40-1.5%	750	0.93	46.6
C40-2.0%	710	0.90	48.8
C60-0.5%	755	0.89	60.1
C60-1.0%	750	0.93	69.6
C60-1.5%	760	0.93	64.9
C60-2.0%	765	0.94	66.1

**Table 5:** Tensile strengths of SFRSCC.

Fiber Volume Fraction (%)	Tensile Strength *σ_t_* (MPa)	
	C40	C60
0.5	14.3 ± 0.3	18.5 ± 0.6
1.0	17.0 ± 0.4	21.4 ± 0.4
1.5	19.8 ± 0.3	23.0 ± 0.5
2.0	23.1 ± 0.5	27.0 ± 0.5

## Data Availability

The data presented in this study are available on request from the corresponding author.
